# Improved NO_x_ Storage/Release Properties of Ceria-Based Lean NO_x_ Trap Compositions with MnO_x_ Modification

**DOI:** 10.3390/ma12132127

**Published:** 2019-07-02

**Authors:** Marcos Schöneborn, Thomas Harmening, Javier Giménez-Mañogil, Juan Carlos Martínez-Munuera, Avelina García-García

**Affiliations:** 1 SASOL Advanced Materials, Anckelmannsplatz 1, 20537 Hamburg, Germany; 2 MCMA Group, Department of Inorganic Chemistry and Institute of Materials, University of Alicante, Carretera Sant Vicent del Raspeig s/n, 03690 Sant Vicent del Raspeig, Alacant, Spain

**Keywords:** ceria, manganese, barium, spinel, lean NOx trap, passive NO_x_ adsorber, NO_x_ storage, NO oxidation

## Abstract

Ceria/spinel-based lean NO_x_ trap compositions with and without barium were modified with MnO_x_ via incipient wetness impregnation. The effect of the MnO_x_ layer on the aged materials (850 °C) as to the NO_x_ storage and release properties was investigated via NO_x_ adsorption (500 ppm NO/5% O_2_/balance N_2_) carried out at 300 °C in a dual-bed with a 1% Pt/Al_2_O_3_ catalyst placed upstream of the samples to generate sufficient amounts of NO_2_ required for efficient NO_x_ storage. Subsequent temperature programmed desorption (TPD) experiments were carried out under N_2_ from 300 °C to 700 °C. The addition of MnO_x_ to the barium free composition led to a slightly reduced NO_x_ storage capacity but all of the ad-NO_x_ species were released from this material at significantly lower temperatures (ΔT ≈ 100 °C). The formation of a MnO_x_ layer between ceria/spinel and barium had a remarkable effect on ageing stability as the formation of BaAl_2_O_4_ was suppressed in favour of BaMnO_3_. The presence of this phase resulted in an increased NO_x_ storage capacity and lower desorption temperatures. Furthermore, NO_x_ adsorption experiments carried out in absence of the Pt-catalyst also revealed an unexpected high NO_x_ storage ability at low NO_2_/NO ratios, which could make this composition suitable for various lean NO_x_ trap catalysts (LNT) related applications.

## 1. Introduction

The implementation of stricter environmental legislations for passenger cars globally, like Euro 6d, US Tier 3 and China 6B calls for improved catalytic systems for emission control [1]. A lot of attention is drawn to the development of improved NO_x_ abatement systems (deNO_x_) for lean-burn engines. As these engines operate at λ > 1, the abatement of NO_x_ requires dedicated technologies, such as selective catalytic reduction (SCR) and lean NO_x_ trap catalysts (LNT), also known as NO_x_ storage/reduction catalysts (NSR) [2,3].

In SCR systems, an aqueous urea solution is injected into the exhaust gas via an onboard-tank leading to the formation of NH_3_, which then reacts with NO_x_ over the catalyst via the standard (1) and fast (2) SCR reactions. Currently, the most common SCR catalysts are metal-substituted zeolites, like Fe-ZSM-5 or Cu-CHA [4].

4NO + 4NH_3_ + O_2_ → 4N_2_ + 6H_2_O (1)

NO + NO_2_ + 2NH_3_ → 2N_2_ + 3H_2_O (2)

The fast SCR reaction (2) is significantly more effective than reaction (1) at low temperatures in the range of 250–300 °C but it requires the presence of NO_2_. Therefore, the SCR catalyst is typically positioned downstream of the diesel oxidation catalyst (DOC), which effectively increases the NO_2_/ NO_x_ ratio [5,6]. Once the NO_2_ concentration exceeds a certain threshold, the NO_2_-SCR reaction (3) also takes place.

4NH_3_ + 3NO_2_ → 7/2N_2_ + 6H_2_O (3)

The NO_x_ abatement mechanism of lean NO_x_ traps is based on alternating lean/rich cycles with long lean phases, in which NO_x_ emissions from the exhaust gases are adsorbed on the catalyst. The stored ad-NO_x_ species are desorbed and reduced to nitrogen on catalytically active noble metals in subsequent short rich periods at elevated temperatures [7]. Most common lean NO_x_ trap catalysts contain a high surface-area material like γ-Al_2_O_3_, mixtures of BaO and CeO_2_ and noble metals, typically Pt or Pd and Rh [8,9].

Barium species, such as BaO or BaCO_3_ act as the primary NO_x_ storage component. They can adsorb large amounts of NO_x_ in the form of surface nitrites and nitrates during lean operation which involves the progressive oxidation from NO to NO_2_ and finally NO_3_^−^. The mechanism of NO_x_ adsorption on barium species has been investigated intensively [10,11,12,13,14,15]. According to these studies, the NO_x_ adsorption takes place via two pathways in parallel. In the “nitrite” route, NO is catalytically oxidized and directly stored on barium sites in form of nitrite ad-species which can be further oxidized to nitrates.

The “nitrate” route is initiated by the catalytic oxidation of NO to NO_2_ (4) which then react on barium sites in a disproportionation reaction, resulting in the formation of nitrate and NO (5).

NO + 1/2O_2_ → NO_2_ (4)

3NO_2_ + BaO → Ba(NO_3_)_2_ + NO (5)

The regeneration of NO_x_ storage sites occurs during rich periods and forms harmless nitrogen via the reduction of NO_x_ species with hydrogen. Cumaranatunge et al. proposed a mechanism that involves the intermediate generation of ammonia [16]. This mechanism has been confirmed in following studies in which various analytical techniques were applied [17,18]. The fundamental chemical reactions can be summarized as:

Ba(NO_3_)_2_ + 8H_2_ → 2NH_3_ + BaO + 5H_2_O (6)

3Ba(NO_3_)_2_ + 10NH_3_ → 8N_2_ + 3BaO + 15H_2_O (7)

The addition of ceria, which has become a major component in catalyst compositions leads to superior NO_x_ storage efficiencies, especially at temperatures below 300 °C [19,20]. Furthermore, ceria plays a substantial role in the water-gas-shift reaction as it provides hydrogen for the regeneration during rich periods and it has a positive impact on the NO_x_ storage/release chemistry due to its interplay with platinum [21,22,23,24]. The mechanisms of NO_x_ storage on ceria under lean conditions involve the formation of various ad-NO_x_ species, such as linear, bidentate and chelating nitrites and nitrates. It was observed that the low-temperature (<100 °C) adsorption of NO on ceria primarily leads to the generation of surface nitrites whereas at higher temperatures nitrates are preferentially formed [19,25,26]. On the contrary, Ryou et al. did not observe the formation of nitrates on Pd/CeO_2_ at 120 °C and they proposed that the presence of water suppresses the oxidation of nitrites [27]. The adsorption of NO_2_ on ceria was studied in detail by Flitschew et al. who combined the well-established diffuse reflectance infrared Fourier transform spectroscopy (DRIFTS) with *in-situ* Raman spectroscopy [28,29]. The authors elaborated that NO_2_ storage on ceria proceeds mainly in two pathways which both lead to the formation nitrate species. The first route involves the adsorption of NO_2_ on cerium(III) sites which are thereby oxidized and transformed to active Ce(IV)-O species (8) which can then react with additional NO_2_ to form nitrates (9). In the second pathway, NO_2_ reacts directly with Ce(IV)-O sites without the contribution of cerium(III) (9).

Ce(III) + NO_2_→ Ce(IV) ⋯ O^2−^+ NO (8)

Ce(IV) ⋯ O^2−^ + NO_2_ → Ce(III) ⋯ O-NO_2_^−^ (9)

Fine-tuning of the regeneration parameters, like temperature and modes of fuel-injection is a demanding task and crucial for the performance such as the long-time durability of the catalyst. Incomplete regeneration inevitably leads to catalyst degradation over time as relevant surface sites remain occupied with adsorbed NO_x_ species. On the contrary, excess H_2_ formed during long rich cycles and low temperatures leads to the generation of high levels of ammonia. This can be a serious concern in LNT-only systems [18,30]. Another important aspect to be considered is the additional fuel-consumption required to enrich the exhaust gas, which eventually leads to increased CO_2_ emissions. Therefore, NO_x_ storage compounds that can effectively be regenerated are urgently needed.

The synergetic combination of passive SCR with LNT catalysts is an efficient solution to attain low NO_x_ tailpipe emissions and to cope with high levels of NH_3_, which may result from the total reduction of NO_x_. This approach has become a widespread and well-studied technology for several years [31,32,33,34,35,36,37,38]. The low-temperature performance of LNT complements SCR and the ammonia generated during the rich regeneration phase can replace or supplement urea required for the SCR reactions.

One major deactivation mechanism of LNT catalysts is the solid acid-base reaction between Al_2_O_3_ and BaO at elevated temperatures resulting in the formation of BaAl_2_O_4_ and thus causing a loss of NO_x_ adsorption sites [39,40]. Improvements in this regard have been reported via the replacement of alumina with the less acidic spinel (MgAl_2_O_4_), leading to the formation of BaAl_2_O_4_ only at higher temperatures [41,42]. In addition, the use of spinel in LNT catalysts has been described to improve the low temperature NO_x_ storage efficiency and to contribute to enhanced SO_x_ tolerance via the formation of sulphates with low thermal stability [43]. 

Another reported deactivation mechanism results from reaction of BaO and CeO_2_ yielding BaCeO_3_, which was detected in LNT formulations prepared by the impregnation of ceria with barium salts [44].

Despite the technological and chemical improvements achieved over the past 20 years, deactivation at elevated temperatures and optimized NO_x_ storage/release properties are perpetual challenges in the design of new formulations for LNT catalysts.

In this regard, the addition of manganese to LNT compositions aiming at improved performance has been proposed and studied by several authors. MnO_x_ –CeO_2_ mixed oxides present high oxygen storage abilities and improved redox properties. They are typically obtained by coprecipitation, sol-gel synthesis or similar routes leading to highs level of homogeneity. The interesting properties predestine these mixed oxides for a substitution of pure ceria in various applications and their superior ability to oxidize soot and NO has already been reported [45,46,47]. Le Phuc et al. observed significantly improved performance of MnO_x_-CeO_2_ containing Pt/Mn-Ce/Ba/Al LNT catalysts in NO_x_ reduction during rich phases, which they attributed to the improved oxygen mobility of the mixed oxide as compared to pure CeO_2_ [48]. 

In another work, Le Phuc studied the contribution of crystalline Mn_2_O_3_ as to the NO_x_ storage performance in Mn/Ba/Al compositions which were investigated in the fresh state, that is, without applying thermal ageing prior to testing [49]. It was found that the manganese sesquioxide only led to improved NO_x_ storage performance in a narrow compositional range whereas higher or lower concentrations had a detrimental effect on the storage efficiency. 

Zhang et al. detected the formation of BaMnO_3_ in the system Pd/Mn/Ba/Al. The presence of this phase led to superior NO oxidation abilities and NO_x_ storage performances of the fresh catalysts [50]. Similar but not identical observations were made by Xiao et al, who noticed the occurrence of BaMnO_3_ only after calcination at 800 °C [51]. This is in accordance with our own investigations, in which we detected this phase in the system Al/Mg/Ce/Mn/Ba after calcination at 850 °C [52].

However, the impact of ageing at elevated temperature as to the NO_x_ storage/release properties of the manganese modified formulations has not been reported yet.

In this work, we present a novel route for the preparation of manganese modified LNT compositions leading to a MnO_x_ layer on ceria/spinel mixtures rather than homogeneous MnO_x_-CeO_2_ mixed oxides. Firstly, the impact on this route in altering the NO_x_ storage/release properties of the resulting spinel/ceria/manganese compositions is investigated. Secondly, the stabilizing function of the MnO_x_ layer as a protective barrier between spinel/ceria and barium is rationalized. The materials described herein were thermally aged at 850 °C prior to testing in order to study their thermal stability, which is a common procedure to simulate catalyst ageing under real conditions [53]. NO_x_ storage experiments were carried in a dual-bed with a 1% Pt/Al_2_O_3_ catalyst placed upstream from the samples. This arrangement mimics a diesel oxidation catalyst, which is typically present in state-of-the-art lean-burn catalyst systems and generates high concentrations of NO_2_. It was shown in previous studies that NO_2_ can be stored much more efficiently than NO on barium and cerium species [19,54].

In order to study the NO oxidation ability provided by the manganese species, additional NO_x_ adsorption experiments were carried out in absence of the Pt/alumina catalyst. 

The thermal stability of the ad-NO_x_ species is important to estimate how efficiently the corresponding NO_x_ storage sites can be regenerated. Therefore, the NO_x_ release properties of our new formulations were investigated in temperature programmed desorption (TPD) experiments. 

## 2. Materials and Methods 

### 2.1. Sample Preparation

MnO_x_ modified carriers with loadings of 9 wt% (calculated as MnO_2_) were prepared by incipient wetness impregnation of homogeneous compositions MgAl_2_O_4_/CeO_2_ (PURALOX MG20 Ce20, commercially available from SASOL) using an aqueous solution of manganese acetate tetrahydrate. The dried samples were then calcined in air at 600 °C.

Both, the Mn-free and the Mn-modified materials were used as starting materials for further wet-impregnation with a barium acetate solution to obtain a loading of approximately 15 wt% BaO. All samples were aged in air at 850 °C for 4 h prior to analyses. Henceforth, the samples are referred to as MgCe (PURALOX MG20 Ce20), MgCe-Mn (MgCe modified with MnO_x_), MgCe-Ba (MgCe modified with BaO) and MgCe-Mn-Ba (MgCe subsequently modified with MnO_x_ and BaO). The chemical compositions of the samples are summarized in Table 1. 

### 2.2. Sample Characterization

Surface area (Brunauer-Emmett-Teller method, BET) and porosity measurements were performed by nitrogen adsorption at −196 °C using a Micromeritics Tri-Star 3000 system. The samples were outgassed overnight at 300 °C under vacuum prior to the measurements. X-ray powder diffraction was conducted on a Phillips X’Pert diffractometer using Cu-Kα radiation (λ = 1.540598 Å). Powder diffractograms were recorded between 5° and 90° (2Ɵ), with a step-width of 0.02°. The sample compositions were determined after digestion in an MLS 1200 microwave apparatus by Inductively Coupled Plasma Atomic Emissions Spectrometer (ICP-OES) using a Spectroflame instrument (SPECTRO). X-ray photoelectron spectra (XPS) were obtained using a K-alpha spectrophotometer (Thermo-Scientific), with a high-resolution monochromator. It comprises a source of electrons and ions for automated load compensation. The X-ray radiation source is equipped with an Al anode (1486.6 eV). The pressure of the analysis chamber was constantly set at 5 × 10^−9^ mbar. The detector was kept in constant energy mode with a pass energy of 200 eV for the survey spectrum and 50 eV for the sweep in each individual region. The binding energy was adjusted using the C-1s transition, appearing at 284.6 eV. Binding energy values measured are accurate to ±0.2 eV. The values of binding energy and kinetic energy were adjusted with the Peak-Fit software of the spectrophotometer. The Mg-1s, Al-2p, Ce-3d, Mn-2p, Ba-3d regions (along with C-1s and O-1s regions) were employed to analyse the surface composition of the carriers in the present study.

### 2.3. NO_x_ Storage/Release Tests

NO_x_ adsorption experiments were conducted in a quartz tubular reactor connected to specific NDIR-UV gas analysers for NO, NO_2_, CO, CO_2_ and O_2_, with the measurement data recorded every 10 seconds. The NO_x_ adsorption (500ppm NO/5%O_2_/balance N_2_) was performed at 300 °C in a dual-bed configuration with a 1% Pt/Al_2_O_3_ commercial catalyst (supplied by Sigma-Aldrich) placed upstream of the sample using a global flow gas of 500 mL/min. The catalyst effectively oxidizes NO to NO_2_, which is required for effective NO_x_ storage. Subsequent temperature programmed desorption (TPD) experiments were carried out under N_2_ from 300 °C to 700 °C (5 °C/min) in order to study the thermal stability of the various stored NO_x_ species. Besides NO_x_ storage ability, this is another important aspect in order to assess the suitability of the different materials for the development of new LNT catalysts.

In addition, NO_x_ adsorption experiments in single-bed configuration were performed without the Pt/alumina catalyst in order to study the NO oxidation ability of the individual samples and to gain further insight into the role of the individual manganese species in the NO_x_ storage processes. 

## 3. Results

### 3.1. Structural Properties

**Figure 1:**
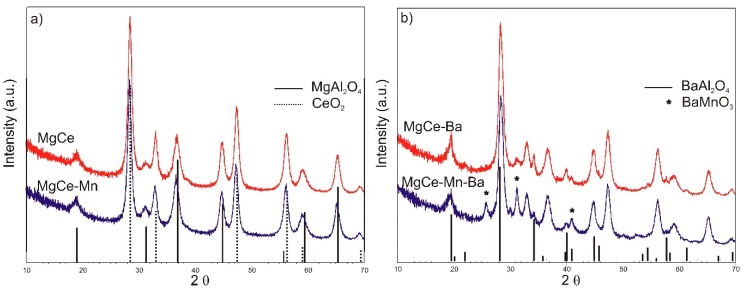
Powder X-ray diffraction (XRD) patterns of (**a**) MgCe and MgCe-Mn (**b**) MgCe-Ba and MgCe-Mn-Ba with simulated reflection positions of MgAl_2_O_4_, CeO_2_, BaAl_2_O_4_ and BaMnO_3_. For better clarity, the reflections of MgAl_2_O_4_ and CeO_2_ are not marked in (**b**).

The phase composition of the samples was analysed by powder X-ray diffraction. It can be seen in Figure 1 that all samples contain MgAl_2_O_4_ and CeO_2_ in crystalline form.

The addition of MnO_x_ to the MgAl_2_O_4_/CeO_2_ composition does not lead to the occurrence of additional reflections in the powder pattern, thus pointing to a homogeneous dispersion of the MnO_x_ species among the surface without the formation of additional crystalline domains detectable by X-ray diffraction (XRD). 

The Ba-containing samples with and without manganese exhibit remarkable differences. In MgCe-Ba, a substantial formation of crystalline BaAl_2_O_4_ is observed. This phase is also present in MgCe-Mn-Ba but the intensity of the relevant reflections is decreased in the X-ray pattern of this material. Most importantly, the formation of BaMnO_3_ is detected in MgCe-Mn-Ba. This phase adopting the perovskite structure is more accurately described as BaMnO_3-__δ_, reflecting the possible formation of vacancies in the oxygen sublattice in conjunction with a partial reduction of manganese (IV). The presence of this phase indicates a close proximity of barium and manganese achieved via the preparation procedure and is in accordance with previous studies, in which the occurrence of BaMnO_3_ in the systems Ba/MnO_x_-CeO_2_ and Mn/Ba/Al_2_O_3_ was also reported [48,50,51,55,56]. No indication of crystalline binary manganese oxides is found in the XRD pattern of MgCe-Mn-Ba. Other authors, however, reported on the occurrence of isolated and crystalline species Mn_2_O_3_ or MnO_2_ in similar systems [49,50]. Remarkable structural and compositional differences of manganese modified supports as a function of the use of either Mn-acetate or Mn-nitrate for preparation were observed and studied by Kapteijn et al., underlining the crucial role of the preparation route as to the resulting phase composition and arrangement [57]. No indication of the presence of BaCeO_3_ is found in either sample, proving that the formation of this phase is thermodynamically unfavoured under the conditions of preparation and ageing. 

Data on the physical properties of all samples is summarized in Table 2. 

Incorporation of about 15% of BaO on the solid precursors by means of wet impregnation followed by aging at 850 °C leads to a significant decrease in BET surface areas and pore volumes of the resulting samples. Nevertheless, the large average pore radius present in all samples is a good indication of their suitability as functional supports for LNT catalysts. 

The analysis of the XPS results (Table 3) becomes complex because the samples suffer from significant levels of carbon contamination due to the presence of carbonates. Carbonation also inevitably occurs under real exhaust conditions. Therefore, purging of the samples via heat treatment in inert gas prior to the NO_x_ storage tests was not applied. Considering the sequential preparation of the samples, both the atomic surface analysis and the metal ratios broadly reflect the decrease in Al, Mg and Ce content after addition of manganese and barium. In addition, the decrease in manganese content after subsequent impregnation with barium is reflected by the XPS results. 

### 3.2. NO_x_ Storage

#### 3.2.1. Dual-bed Experiments

The results of the NO_x_ adsorption tests measured in dual-bed configuration are displayed in Table 4. 

The total NO_x_ retention capacity expressed as mmol of NO_x_ stored/m^2^ of carrier follows the order—MgCe-Mn < MgCe < MgCe-Ba << MgCe-Mn-Ba. It is worth mentioning that the surface areas of the samples with barium are significantly lower, resulting in a different order of NO_x_ retention capacity if expressed as mmol of NO_x_ stored/g of carrier—MgCe-Mn < MgCe-Ba < MgCe << MgCe-Mn-Ba. This is of importance as the reduction in surface area upon Ba-addition and ageing clearly contribute to the deactivation of the support in terms of NO_x_ storage ability. In order to study the role of the different species present in the samples, the surface area related expression is applied in this work.

In the case of the Ba-free samples, the presence of MnO_x_ slightly reduces the NO_x_ storage capacity, thus illustrating that the MnO_x_ as such does not contribute to the NO_x_ storage process at all or only to a negligible extend. Apparently, relevant NO_x_ storage sites of ceria and spinel are partially occupied and thus inactivated by Mn-species. This can also be deduced from the XPS analyses revealing a lower surface concentration of cerium and also a higher surface concentration of carbon species in the MnO_x_ modified samples. MgCe-Ba (less cerium and magnesium but barium on the surface) shows a marked but unexpected small increase in NO_x_ retention capacity compared to MgCe, which indicates that only a small amount of unreacted (and X-ray invisible) BaO or BaCO_3_ is still present after ageing. MgCe-Mn-Ba on the contrary, exhibits superior NO_x_ storage ability, pointing to a significant stabilizing and hence beneficial effect of MnO_x_ on the Ba-assisted NO_x_ storage process. The introduction of MnO_x_ as protective layer between BaO and spinel/ceria leads to the formation of BaMnO_3_ and reduces the amount of undesired BaAl_2_O_4_ generated after ageing, as observed in the XRD pattern. Thus, the occurrence of the perovskite phase most likely accounts for the high retained NO_x_ storage ability after ageing. 

#### 3.2.2. Single-bed Experiments

**Figure 2:**
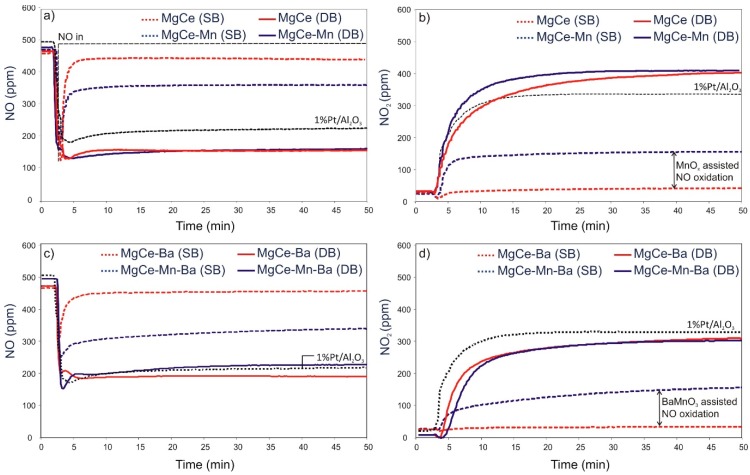
Profiles of NO and NO_2_ of during NO_x_ adsorption at 300 °C under single-bed (SB) and dual-bed (DB) conditions. (**a**) NO and (**b**) NO_2_ profiles of MgCe and MgCe-Mn. (**c**) NO and (**d**) NO_2_ profiles of MgCe-Ba and MgCe-Mn-Ba. Profiles of Pt/alumina are included for comparison.

Single-bed experiments without the Pt/alumina catalyst were conducted to study the effect of Mn-addition on NO oxidation ability. The absence of the noble metal catalyst is required as its outstanding oxidation capacity does not allow for a proper investigation of the NO oxidation contribution of the carriers. Table 5 compiles the NO and NO_2_ levels quantified for the samples MgCe and MgCe-Mn during NO_x_ adsorption in single-bed experiments. The adsorption curves obtained for MgCe and MgCe-Mn observed in single-bed (SB) and dual-bed (DB) experiments are shown in Figure 2). 

MgCe-Mn presents a significantly higher NO_2_ production than MgCe evident from the NO_2_/NO ratios obtained in single-bed experiments, revealing its superior NO oxidation activity promoted by MnO_x_ addition. The ability of binary manganese oxides, especially Mn_2_O_3_, to oxidize NO has also been observed and described in detail by Guo et al. [58]. Although the analysis of the oxidation state of manganese is not part of this study, the presence of highly dispersed Mn_2_O_3_ species in MgCe-Mn therefore appears to be a valid assumption.

**Figure 3:**
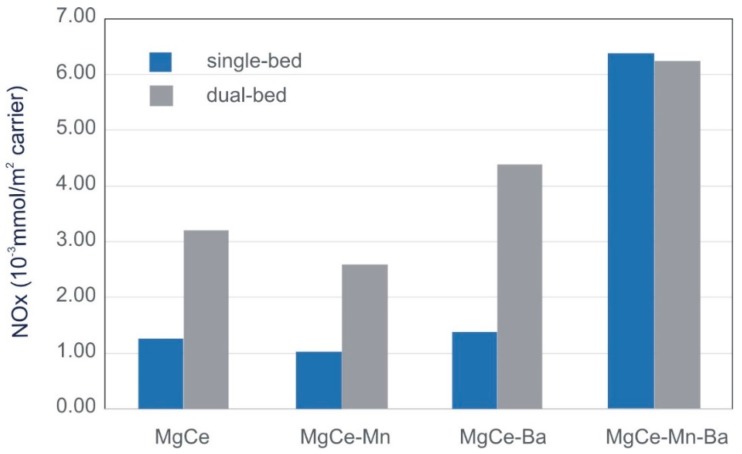
Stored amounts of NO_x_ observed in single-bed and dual-bed experiments.

Although the difference in NO_2_/NO ratios between MgCe-Ba and MgCe-Mn-Ba is in the same range as in the Ba-free samples, a remarkable higher NO_x_ storage ability is found in MgCe-Ba-Mn in the single-bed experiments. The amount of stored NO_x_ is in the same range as observed in dual-bed experiments and is around 4.5 times higher than that observed for MgCe-Ba. The amounts of stored NO_x_ observed in single-bed and dual-bed experiments are summarized in Figure 3. The NO and NO_2_ adsorption progressions shown in Figure 2c,d prove that the high NO_x_ storage efficiency of MgCe-Mn-Ba results from the effective NO_2_ production in conjunction with the availability of storage sites. NO as such is only adsorbed to a negligible extend. The value of Ba-Mn interactions for fast NO_x_ storage has previously been reported by Zhang et al. [50]. They have found that the NO oxidation ability of Mn-sites in close proximity to NO_x_ storage sites of barium leads to very high NO_x_ storage efficiencies. It can be speculated that the perovskite BaMnO_3_, which accommodates manganese in the rather high oxidation state +IV, combines both, high redox potential and efficient NO_x_ storage sites in one solid. The high activity of BaMnO_3_ for NO and soot oxidation has also been pointed out by Gao et al. [55]. 

### 3.3. NOx Release

#### 3.3.1. TPD after NO_x_ Adsorption under Dual-bed Configuration

**Figure 4:**
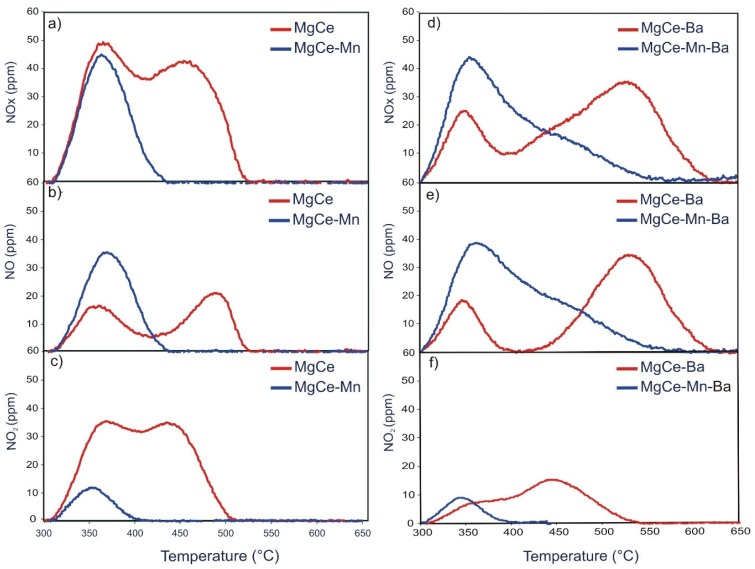
Temperature programmed desorption (TPD) profiles (dual-bed configuration) of MgCe and MgCe-Mn (**a**) NO_x_, (**b**) NO (**c**) NO_2_ and MgCe-Ba and MgCe-Mn-Ba (**d**) NO_x_, (**e**) NO (**f**) NO_2_.

The temperature programmed desorption (TPD) of NO_x_, NO and NO_2_ after NO/O_2_ retention under dual-bed configuration was analysed in the temperature range of 300–700 °C. Figure 4 shows the TPD profiles of the different samples. The shape of the TPD profile of MgCe with two maxima—the first one centred at about 360 °C and the second centred at about 450 °C—indicates that there are two main ad-NO_x_ species present on this sample. The individual contribution of NO and NO_2_ with the quantification given in Table 6 reveals that a significant fraction of the stored NO_x_ is released from this sample in the form of NO_2_. The NO_2_ desorption takes places in a wide temperature window between 300 and 510 °C. The release of NO takes place in two steps, the first centred at about 350 °C and the second centred at about 490 °C. 

The presence of barium leads to distinct changes in the TPD profiles in MgCe-Ba and MgCe-Mn-Ba. Significantly more NO is released from MgCe-Ba than from MgCe with the concentration of NO_2_ being almost identical. Similar to MgCe, the NO profile of MgCe-Ba shows two peaks. The first one is centred at about 350 °C and shows a comparative shape and peak area than the corresponding peak in MgCe. The second peak is centred at about 530 °C, with a much larger peak area. The NO release is taking place between 440 °C and 610 °C, so that the maximum desorption temperature of NO is increased by about 120 °C compared to MgCe. The concentrations and release temperatures of NO_2_ are very similar for MgCe and MgCe-Ba although the main contribution of NO_2_ in MgCe-Ba originates from the desorption processes taking place between 400 °C and 520 °C. 

The observations made in the TPD profiles of MgCe and MgCe-Ba lead to the conclusion that the active barium species form comparatively strongly bound ad-NO_x_ species, which mainly form NO upon decomposition. This is in line with studies from Lietti et al. [11,12], who reported that NO is the main decomposition product of NO_x_ species stored on barium, with TPD profiles very similar to the high temperature peak shown in Figure 4e. This furthermore supports the assumption (see Section 3.2.1) that there are still active barium species left on MgCe-Ba although only BaAl_2_O_4_ can be detected in the X-ray pattern.

The presence of MnO_x_ forming a layer on spinel/ceria displays unexpected substantial effects on the NO_x_ release chemistry of the samples with and without BaO. The NO_x_ desorption is dominated by NO in the Mn-containing samples with only minor contribution of NO_2_ in marked contrast to the Mn-free samples. In the case of the Mn-Ba combination, an additional contribution of NO species released in the range of 420–520 °C is observed, thus pointing to the generation of additional storage sites. This contribution can be linked to the presence of BaMnO_3_ since this desorption peak is absent in MgCe-Mn. Secondly, the NO_x_ release process is much more efficient in the MnO_x_ modified samples, in particular in MgCe-Mn. As it can be seen in Figure 4a–c, the NO_x_ desorption is taking place in the narrow range 300–450 °C. The high temperature desorption of NO and NO_2_ is completely absent in the MnO_x_ modified sample. A similar observation is made in the TPD profile of MgCe-Mn-Ba, in which the decreased fraction of NO_x_ desorbed at higher temperatures upon manganese modification is also apparent. 

#### 3.3.2. TPD after NO_x_ Adsorption under Single-bed Configuration

**Figure 5:**
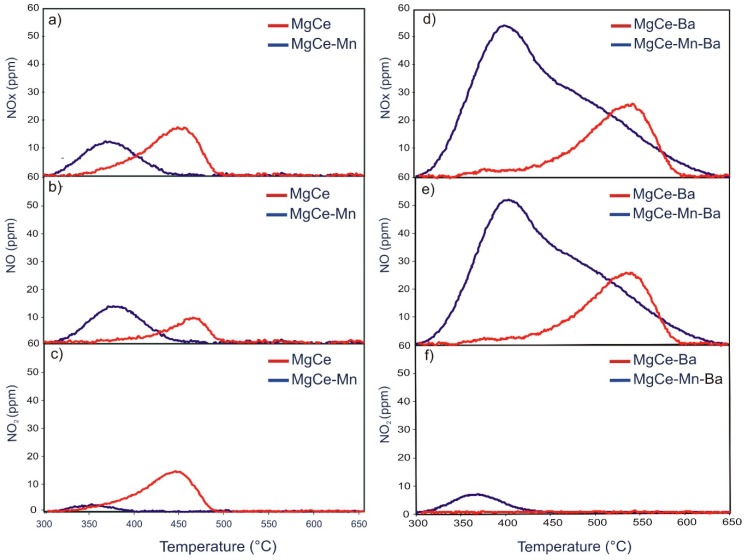
TPD profiles (single-bed configuration) of MgCe and MgCe-Mn (**a**) NO_x_, (**b**) NO (**c**) NO_2_ and MgCe-Ba and MgCe-Mn-Ba (**d**) NO_x_, (**e**) NO (**f**) NO_2_.

Effects induced by MnO_x_ addition are even more pronounced in the TPD profiles of MgCe and MgCe-Mn after NO_x_ storage under single-bed configuration (Figure 5 and Table 7).

The majority of stored NO_x_ species is released from MgCe in the range of about 320–490 °C in the form of NO_2_. The asymmetry of the NO_2_ peak indicates the presence of differently bound ad-NO_x_ species with the main population being desorbed at the peak maximum of about 440 °C. 

TPD profiles of MgCe-Mn show a significant increase in NO desorption whereas the concentration of released NO_2_ is close to zero. The high-temperature desorption peak of NO detected in MgCe is not present in MgCe-Mn but a new one is observed ranging from 300–460 °C. This can be understood in conjunction with the dual-bed experiments, see Section 3.3.1, Figure 4b. Upon the addition of manganese, the low-temperature NO peak gains intensity whereas the high-temperature peak disappears. The absence of the high-temperature NO peak and the low concentration of detectable NO_2_ in MgCe-Mn are in accordance with the dual-bed experiments and support the theory of inhibited ceria/spinel sites by manganese species. 

The NO_x_-TPD profile of MgCe-Mn-Ba is very similar to the profile obtained after adsorption in dual-bed experiments whereas the low-temperature peak associated with NO release is absent in the profile of MgCe-Ba, thus illustrating the similarity of ad-NO_x_ species formed on BaMnO_3_ with and without the platinum oxidation catalyst. The presence and intensity of the low-temperature NO peaks of MgCe-Mn and especially MgCe-Mn-Ba demonstrate that the corresponding ad-NO_x_ species are only formed if considerable amounts of NO_2_ are available in the feed gas or in other words: if an effective NO → NO_2_ oxidant is present.

## 4. Discussion

The effect of MnO_x_ addition to LNT compositions based on MgAl_2_O_4_/CeO_2_ on the NO_x_ storage/release properties was investigated at an adsorption temperature of 300 °C. The results clearly show that both, compositions with and without barium, can be improved significantly by the formation of a MnO_x_ layer on the spinel/ceria mixture. The advantages of adding manganese are different in both type of materials indicating that at least two directions in the development of novel LNT catalysts may be pursued. 

1. Barium-free compositions. The formation of a manganese oxide layer on MgAl_2_O_4_/CeO_2_ primarily alters the chemical nature of the generated ad-NO_x_ species, as they exhibit lower thermostabilities than those generated without Mn-addition. This approach is clearly different from the formation of MnO_x_-CeO_2_ mixed oxides, which is described in several studies [45,46,47]. 

The presence of the MnO_x_ layer significantly narrows the temperature range, in which the full amount of stored NO_x_ is desorbed and lowers the maximum desorption temperature by about 100 °C. This finding makes the manganese modified composition an appealing candidate for the development of passive NO_x_ adsorbers (PNA). In contrast to conventional LNT catalysts, passive NO_x_ adsorbers are regenerated at elevated temperatures without additional rich pulses. This approach has already been described in the early 2000s [59,60] but has gained particular attention just recently due to the stricter legal requirements for NO_x_ emissions and the implementation of the more realistic and challenging test procedure WLTP (Worldwide Harmonized Light-Duty Vehicles Test Procedure) for type approval. PNAs in combination with passive SCR catalysts can effectively improve the cold start NO_x_ conversion at temperatures lower than 200 °C without causing additional fuel penalties [61]. The performance of PNAs is determined by (a) the ability to store NO_x_ at low temperatures of about 150 °C and (b) effective NO_x_ release in a narrow and low temperature range, so that the conversion over the SCR catalyst such as the recovery of NO_x_ storages sites can take place efficiently. Ceria- and manganese-based PNAs have already been described in literature but our newly developed composition MgAl_2_O_4_/CeO_2_ with MnO_x_ appears to be an interesting and sophisticated alternative due to its narrow NO_x_ release window from 300–450 °C [62,63]. Although the observed NO_x_ release behaviour is very promising, the assessment of the low-temperature NO_x_ storage efficiency has not been investigated yet and is part of ongoing studies. 

2. Compositions with barium. In the case of Mn-free sample, BaAl_2_O_4_ is formed upon ageing at 850 °C, evidently leading to a decrease in NO_x_ storage sites. The introduction of a protective layer of MnO_x_ between spinel/ceria and barium beneficially suppresses the formation of BaAl_2_O_4_ in favour of BaMnO_3._ This perovskite phase accommodating manganese (IV) was proven to be very active for NO_x_ storage even at low NO_2_/NO ratios. This beneficial effect was not observed in former investigations of Ba/Mn/alumina compositions prepared by a sequential impregnation of γ-Al_2_O_3_ with barium and manganese salts followed by calcination at only 500 °C, not leading to the formation of BaMnO_3_ [49]. This illustrates that the temperature treatment along with the resulting phase composition has a crucial impact on the resulting NO_x_ storage properties. The NO_x_ retention ability and the NO_x_ release mechanisms of the manganese modified material do not change if the sample is positioned downstream of an external Pt-catalyst. This is of enormous relevance as state of the art barium-based LNT catalysts require high levels of NO_2_ for effective NO_x_ conversion [54]. The observation suggests that LNT catalysts based on this formulation can tolerate a reduced noble metal (Pt/Pd) content without losing performance. Furthermore, these materials offer the opportunity for the development of new deNO_x_ concepts, in which the LNT function is not mandatorily placed downstream of the DOC function or in which the DOC requires a certain level of NO_x_ retention ability itself.

A detailed understanding of the chemistry involved in the generation of the various ad-NO_x_ species presenting different thermostabilities could be gained by *operando* DRIFTS measurements in combination with Raman spectroscopy and other sophisticated techniques. These investigations might provide a complementary overview on the diverse processes taking place on the surface of the materials during NO_x_ storage and release. The individual contributions of the different compounds present in the compositions could be elucidated and rationalized this manner. These studies are in progress and will be presented in following publications.

## 5. Patents

WO2016/142058A1 (SASOL Germany GmbH).

## Figures and Tables

**Table 1:** Chemical composition (wt%) of the samples determined by Inductively Coupled Plasma (ICP) analysis.

Sample	Al_2_O_3_	MgO	CeO_2_	MnO_2_	BaO
MgCe	63.0	17.0	20.0	0	0
MgCe-Mn	58.3	14.9	17.7	9.1	0
MgCe-Ba	52.8	14.7	17.0	0	15.5
MgCe-Mn-Ba	48.4	13.3	15.5	7.3	15.5

**Table 2:** Physical properties of the samples studied in this work.

Sample	S_BET_ (m^2^/g)	r_P_ (nm)	V_P_ (cm^3^/g)
MgCe	96	13	0.64
MgCe-Mn	104	10	0.53
MgCe-Ba	68	11	0.37
MgCe-Mn-Ba	59	11	0.31

**Table 3:** Surface atomic contents and ratios estimated by X-ray photoelectron spectroscopy (XPS).

Element	Sample			
	MgCe	MgCe-Mn	MgCe-Ba	MgCe-Mn-Ba
C	33.46	43.7	50.4	58.1
O	40.0	37.7	35.5	30.1
N	0.4	0.3	0.7	0.5
Al	17.3	11.1	10.5	8.5
Ce	2.9	2.5	0.7	0.5
Mg	2.3	2.3	1.1	1.0
Mn	0.0	2.5	0.0	0.7
Ba	0.0	0.0	1.2	0.8
Al/(Al + Ce + Mg + Mn + Ba) ^1^	0.77	0.60	0.78	0.74
Ce/(Al + Ce + Mg + Mn + Ba) ^1^	0.13	0.14	0.05	0.04
Mg/(Al + Ce + Mg + Mn + Ba) ^1^	0.10	0.13	0.08	0.09
Mn/(Al + Ce + Mg + Mn + Ba) ^1^	-	0.13	-	0.06
Ba/(Al + Ce + Mg + Mn + Ba) ^1^	-	-	0.09	0.07

^1^ The ratios were calculated based on the content of the elements.

**Table 4:** Amounts of NO_x_ stored on the samples in dual-bed experiments.

Samples without Ba	NO_x_ Stored (10^−3^ mmol/m^2^_carrier_)	Samples with Ba	NO_x_ Stored (10^−3^ mmol/m^2^_carrier_)
MgCe	3.23	MgCe-Ba	4.41
MgCe-Mn	2.60	MgCe-Mn-Ba	6.27

**Table 5:** NO_2_/NO ratios and amounts of NO_x_ stored on the different samples obtained under single-bed configuration.

Sample	NO_2_/NO Ratio	NO_x_ Stored (10^−3^ mmol/m^2^_carrier_)
MgCe	0.07	1.27
MgCe-Mn	0.43	1.03
MgCe-Ba	0.08	1.40
MgCe-Mn-Ba	0.48	6.39

**Table 6:** Released amounts of NO_x_, NO and NO_2_ after adsorption under dual-bed configuration.

Sample	NO_x_ Released (10^−3^ mmol/m^2^_carrier_)	NO Released (10^−3^ mmol/m^2^_carrier_)	NO_2_ Released (10^−3^ mmol/m^2^_carrier_)
MgCe	2.47	0.79	1.68
MgCe-Mn	0.97	0.78	0.19
MgCe-Ba	4.69	3.15	1.54
MgCe-Mn-Ba	4.76	4.39	0.37

**Table 7:** Released amounts of NO_x_, NO and NO_2_ after adsorption under single-bed configuration.

Sample	NO_x_ Released (10^−3^ mmol/m^2^_carrier_)	NO Released (10^−3^ mmol/m^2^_carrier_)	NO_2_ Released (10^−3^ mmol/m^2^_carrier_)
MgCe	0.59	0.18	0.41
MgCe-Mn	0.36	0.32	0.04
MgCe-Ba	1.34	1.34	0
MgCe-Mn-Ba	5.05	4.61	0.44
